# 239 The Influence of role models on participation in sport and physical activity among young males and females in Ireland

**DOI:** 10.1093/eurpub/ckae114.028

**Published:** 2024-09-26

**Authors:** Eimear Kelly, Katie Liston, Kieran Dowd, Aoife Lane

**Affiliations:** Department of Sport and Health Sciences in Technological University of the Shannon: Midlands Midwest, Athlone, Ireland; Social Sciences of Sport, School of Sport, Ulster University, Belfast, Ireland; Department of Sport and Health Sciences in Technological University of the Shannon: Midlands Midwest, Athlone, Ireland; Department of Sport and Health Sciences in Technological University of the Shannon: Midlands Midwest, Athlone, Ireland

## Abstract

**Purpose:**

The aim of this study is to describe the influence of role models (RMs) on sport participation (SP) and physical activity (PA) levels in adolescent males and females across Ireland, and specifically to understand sex specific differences given that young males are typically more active and play more sport than their female counterparts.

**Methods:**

Two sporting role model questions were included in the 2022 All Island Children’s Sport Participation and Physical Activity Study (CSPPA) Study. The sample included 5,815 participants (aged 10-19 years) from primary (N = 2,265) and post primary (N = 3,551) schools. Binary logistic regression was used to determine the relationship between RMs, such as parents, coaches, and sports stars with PA and SP.

**Results:**

The majority (84%) of youth are not meeting PA guidelines, with males more sufficiently physically active (20% v 11.9%) than females. Weekly SP (52.4% v 47.6%) and sport club membership (69.6% v 65.4%) is also higher in males. The most popular sporting RMs across all youth are sports stars, coaches, Dad’s and friends with sports stars, Dad’s and coaches most influential on PA and SP. Male and female youth with sports star RMs were on average twice as likely to meet PA guidelines and play sport weekly (p<.05), the latter stronger for males. All youth appear to select sports star RMs in the sport they play, while males are more likely to choose male and international sports stars and females are equally likely to choose male and female, and Irish rather than international sports stars as RMs.

**Conclusions:**

Role models, particularly sports stars, appear to influence SP and PA in Irish youth with some differences across gender. Young people select ‘relevant’ sport stars as RMs in terms of sport and gender, with the latter more notable for males perhaps suggesting a lack of visibility and availability of female sport stars. Given the policy context and potential impact of sports stars on SP and PA, there is an opportunity to further understand and leverage how young females in particular select and engage with sport stars to increase SP and PA.

**Support/Funding Source:**

TUS President Doctors Scholarship

